# Supplementary material for: A biogenic secondary organic aerosol source of cirrus ice nucleating particles
Source: Nat Commun. 2020 Oct 1;11:4834. doi: 10.1038/s41467-020-18424-6 (PMC7529764; doi:10.1038/s41467-020-18424-6)
Supplement: Supplementary file 1 — Supplementary Information [file 41467_2020_18424_MOESM1_ESM.pdf]

Supplementary Information

A Biogenic Secondary Organic Aerosol Source of Cirrus Ice Nucleating Particles

Wolf et al. 2020

Supplementary Methods

Station Meteorology

Meteorological variables are averaged and reported in five-minute time intervals. The time series of ambient temperature, barometric pressure, relative humidity, wind speed, wind direction, and cloud liquid water content (LWC) are illustrated in Supplementary Figure 1b. The average values of these variables during INP sampling are plotted against INP concentration in Supplementary Figure 1a during 10-minute and daily averaged sampling periods. We have also calculated ordinary least squares linear regressions on INP concentration and meteorology. Regression coefficients and p-values for both 10 minute and daily sampling periods are recorded in Supplementary Table 1. Supplementary Table 1 contains the regression coefficients of linear fits to the data in Supplementary Figure 1b.

INP Size Derivation from the Concentration Enhancement Factor

INPs were alternatively sampled directly from the whole air inlet and from a virtual impaction aerosol concentrator, the Portable Fine Particle Concentrator (PFPC)[1]. The Whole Air Inlet operates with a 50% cut-of diameter of approximately 30 microns making them capable of sampling both dry aerosol particles and cloud droplets. The PFPC concentration enhancement factor is size dependent, as smaller particles are less effectively concentrated via the virtual impaction technique than larger particles[1]. Average INP diameter can thereby be derived by comparing the INP-specific enrichment factor to an aerosol-enhancement factor calibration curve. Such calibration was made by comparing total particle concentrations as a function of size before and after the PFPC using a scanning mobility particle sizer (SMPS) coupled with a TSI condensation particle counter (TSI 3010). A total of three calibrations were performed during this study by sampling and averaging particle concentrations on and off the concentrator over 10 minutes. Due to variability in the concentration enhancement factor, application of the aerosol calibration curve to the INP enhancement factor introduces an uncertainty in average INP size (Supplementary Figure 2).

INP Correlations

INP concentration and diameter correlate with ambient concentration of small ($D < 150$ nm) particles and non-refractory organic mass fraction. Particle concentration is derived from SMPS size distributions, and organic mass fraction is derived from ACSM data.

Station Gas Phase Chemistry

Gas phase chemistry data are averaged and reported in five-minute time intervals. The time series of ambient ozone (O_3), sulfur dioxide (SO_2), nitric oxide (NO), and nitrogen dioxide (NO_2) concentrations are illustrated in Supplementary Figure 4b. The 10 minute and daily average values of these variables during INP sampling are plotted against 10 minute and daily average INP concentration in Supplementary Figure 4a. Supplementary Table 2 contains the regression coefficients and their p-values of ordinary least squares linear regressions to the data in Supplementary Figure 4a.

Station Bulk Aerosol Chemistry

Mass loading and chemical composition of bulk $PM_{1.0}$ non-refractory aerosol was measured using a Time of Flight Aerosol Chemical Speciation Monitor (ToF-ACSM; Aerodyne Research Inc.). Average mass loading of total organic aerosol and inorganic species including sulfate (SO_4^{2-}

), ammonium (NH_4^+), nitrate (NO_3^-), and chlorine (Cl^-) were averaged over each sampling period for comparison to the offline filter sample chemical analysis. There is no significant correlation between average bulk aerosol composition and depositional INP concentration (Supplementary Figure 5). Supplementary Table 4 contains the regression coefficients of linear fits to the data in Supplementary Figure 5. Regression coefficients do not improve when time resolution is increased from daily to 10-minute sampling averages.

SOA Viscosity, Water Diffusion, and Glassy State Phase Transition Calculations

The glass transition temperature and viscosity of the 2-MT OS at RH=84% are estimated based on the method described by previous studies[2–4]. Since no hygroscopicity measurements have been performed on 2-MT OS to our knowledge, we use the hygroscopicity of other biogenic organosulfates – limonene-derived organosulfate (L-OS) – as a surrogate for 2-MT OS. L-OS has a hygroscopicity parameter (κ) value of 0.03 for 100 nm particles at ~85% RH[5].

The viscosity of IEPOX-derived OS is calculated based on a modified version of the Vogel-Tammann-Fulcher (VTF) equation (Eqs. S1 & S2) by Angell et al[6,7].

$$\eta\,(RH)=\eta_{\infty} e^{\frac{T_0 D}{T-T_0}} \tag{S1}$$

where η_{∞} is viscosity at infinite temperature and assumed to be 10^{-5} Pa s, T_0 is the Vogel temperature, T is the ambient temperature, and D is the fragility parameter that controls how closely a material follows the Arrhenius law[6]. When T reaches T_g, η reaches 10^{12} Pa s, a value commonly associated with glass transition. Then Eq. (1) becomes

$$T_g/T_0 = 1 + 0.0255D \tag{S2}$$

We then apply the Gordan-Taylor mixing rule to calculate the T_g of the 2-MT OS and water mixture. The Gordon-Tayler constant between IEPOX-OS and water, k_{GT}, is assumed to be 2.5 based on previous studies[4]. The glass transition temperatures of 2-MT OS at dry conditions and water are notated as $T_{g,\,dry}$ and $T_{g,\,water}$ (136 K). The mass fraction of the 2-MT OS in the whole mixture is $w_{org}(RH)$ and is a function of relative humidity (RH).

$$T_{g,\,mix}(RH) = \frac{\left(1 - w_{org}(RH)\right)T_{g,\,water} + \frac{1}{k_{GT}} w_{org}(RH)\; T_{g,dry}}{\left(1 - w_{org}(RH)\right) + \frac{1}{k_{GT}} w_{org}(RH)} \tag{S3}$$

The mass fraction of 2-MT OS at any specific RH can be estimated based on effective hygroscopicity parameter (κ):

$$m_{H_2O} = \frac{\kappa \rho_w m_{2\text{-MT OS}}}{\rho_{2\text{-MT OS}}\left(\frac{1}{a_w} - 1\right)} = \frac{\kappa \rho_w m_{2\text{-MT OS}}}{\rho_{2\text{-MT OS}}\left(\frac{100}{RH} - 1\right)} \tag{S4}$$

where m_{H_2O} and $m_{2\text{-MT OS}}$ are the masses of water and 2-MT OS. ρ_w and $\rho_{2\text{-MT OS}}$ are the densities of water (1 g cm^{-3}) and 2-MT OS (assumed to be 1.2 g cm^{-3}). The water activity and the relative humidity are a_w and RH, respectively.

Based on Eqns. (S1) through (S4) and the parameters chosen for 2-MT OS (T_g=276 K, k_{GT}=2.5, κ=0.03, D=10), the glass transition temperature of 2-MT OS is estimated to be 237 K (36 ºC). This temperature is warmer than the conditions at which SPIN operated (–46 ˚C). By including the uncertainty of D and κ values (D=10(20), κ=0.02(0.05)) as lower and upper bounds), the glass transition temperature of 2-MT OS at SPIN's operating conditions (T = –46 ºC, RH_{ice} = 130%) is estimated to be 237^{+10}_{-16} K and the viscosity is estimated to be $10^{12^{+0}_{-2}}$ Pa s.

The diffusion timescale of water within the 2-MT OS particle can be calculated as follows. The viscosity of 2-MT OS was estimated to be $10^{12^{+0}_{-2}}$ Pa s at the cirrus conditions, which is equivalent to a calculated diffusion coefficient of $10^{-24^{+2}_{-0}}$ m^2 s^{-1} using the Stokes-Einstein equation. Based on the findings by Price et al., the water diffusion rate is ~$10^{-20^{+2}_{-0}}$ m^2 s^{-1}. The timescale for water to diffuse through a 100-nm particle is estimated to $2\times10^{8^{+0}_{-2}}$s based on the equation provided by Renbaum-Wolff et al.[7]. This is longer than the timescale typical for ice nucleation[8].

These results demonstrate that the glass transition temperature of 2-MT OS was likely warmer than our experimental conditions (–46 ˚C), thereby allowing the particles remain glassy or semi-solid. This estimation supports our conclusion that 2-MT OS could remain sufficiently viscous to promote heterogeneous ice nucleation at cirrus conditions.

Supplementary Figures

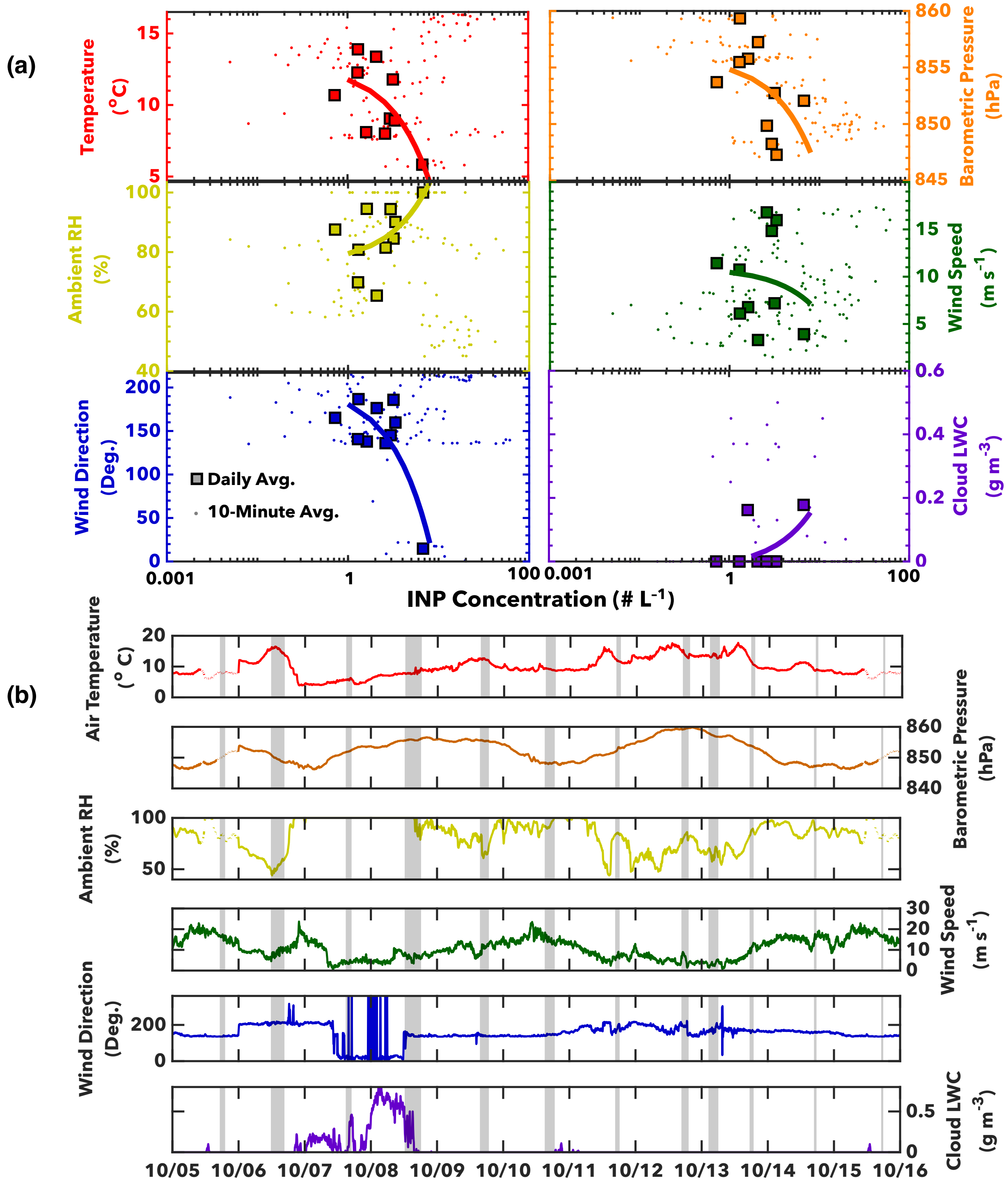

Supplementary Figure 1. Puy de Dôme observatory meteorology. (**a**) Variables plotted against INP concentrations. Each data point represents the average within a 10-minute or daily INP sampling period. Trendlines indicate linear regressions of daily averaged values. (**b**) Time series of station meteorology. Shaded regions correspond to periods of INP measurements.

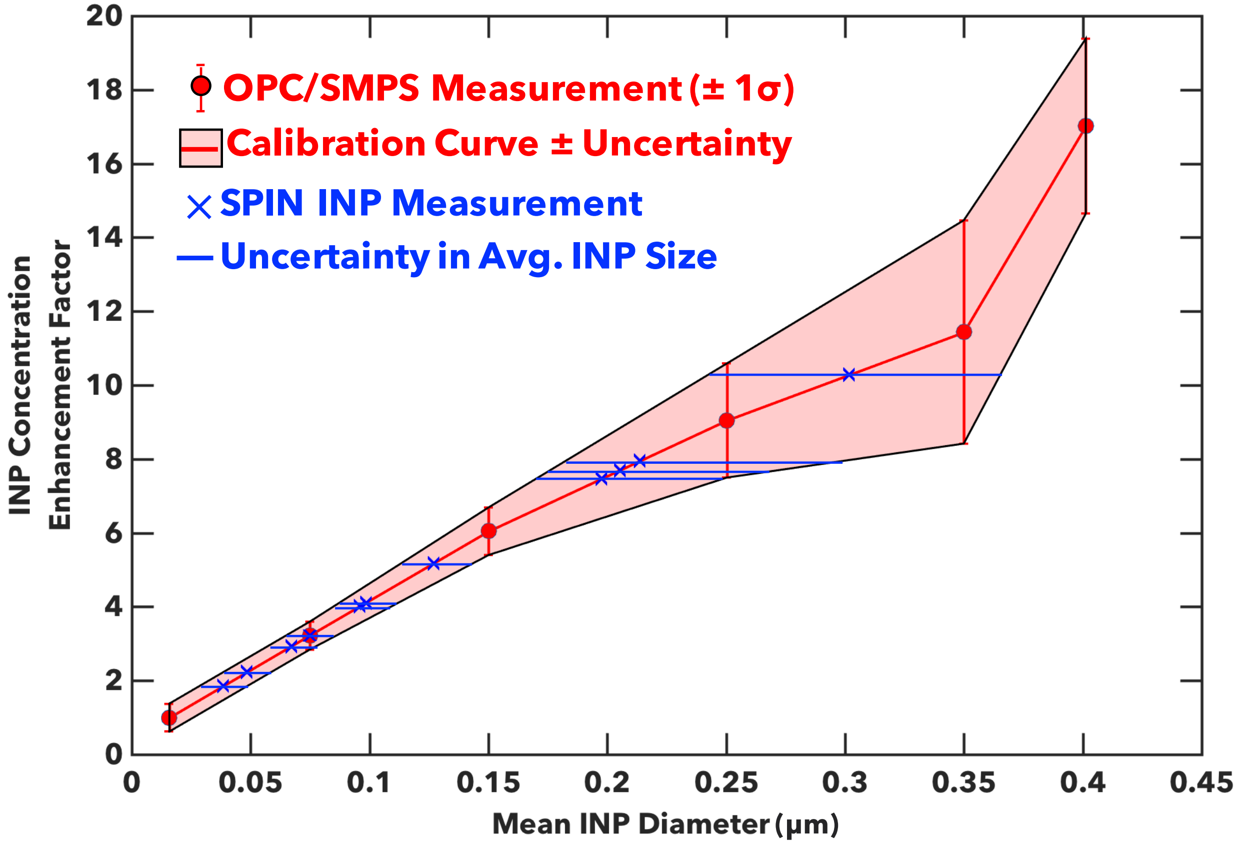

Supplementary Figure 2. INP size derivation from concentrator enhancement factor. Red data correspond to calibration of total aerosol concentration enhancement. Vertical error bars represent a standard deviation of variability in aerosol concentration enhancement. Blue data correspond to each sampling day's average depositional INP concentration enhancement. Horizontal error bars illustrate uncertainty in mean INP diameter resulting from uncertainty in the calibration. The particle enrichment factor plateaus at 25 for particles greater than 0.45 μm.

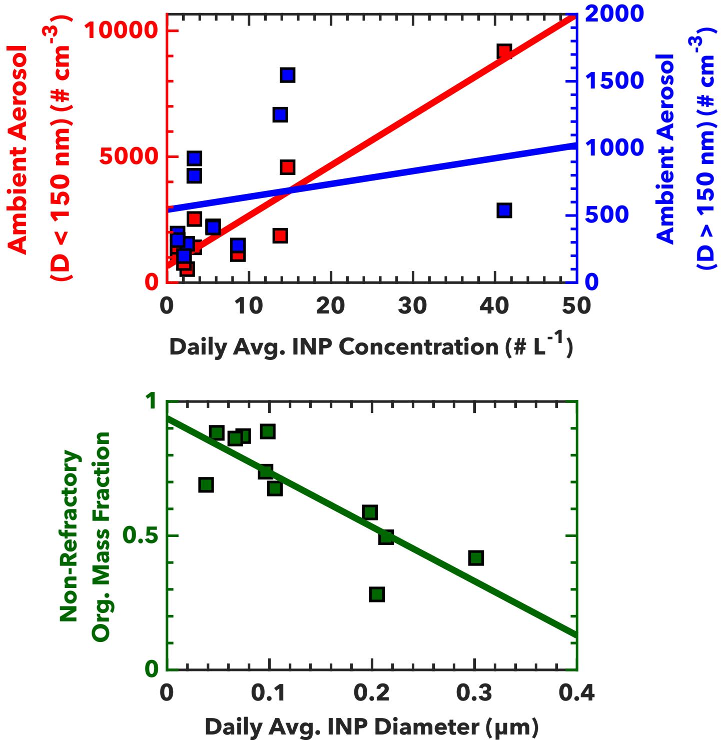

Supplementary Figure 3. Correlations between [INP], size, and organic mass fraction. Data points indicate daily average [INP], ambient aerosol concentration, and ACSM-derived non-refractory organic mass fraction from Oct. 5th to Oct. 15th, 2018. Trendlines indicate ordinary least-squares linear regressions. Statistical variables for these regressions are tabulated in Supplementary Table 2.

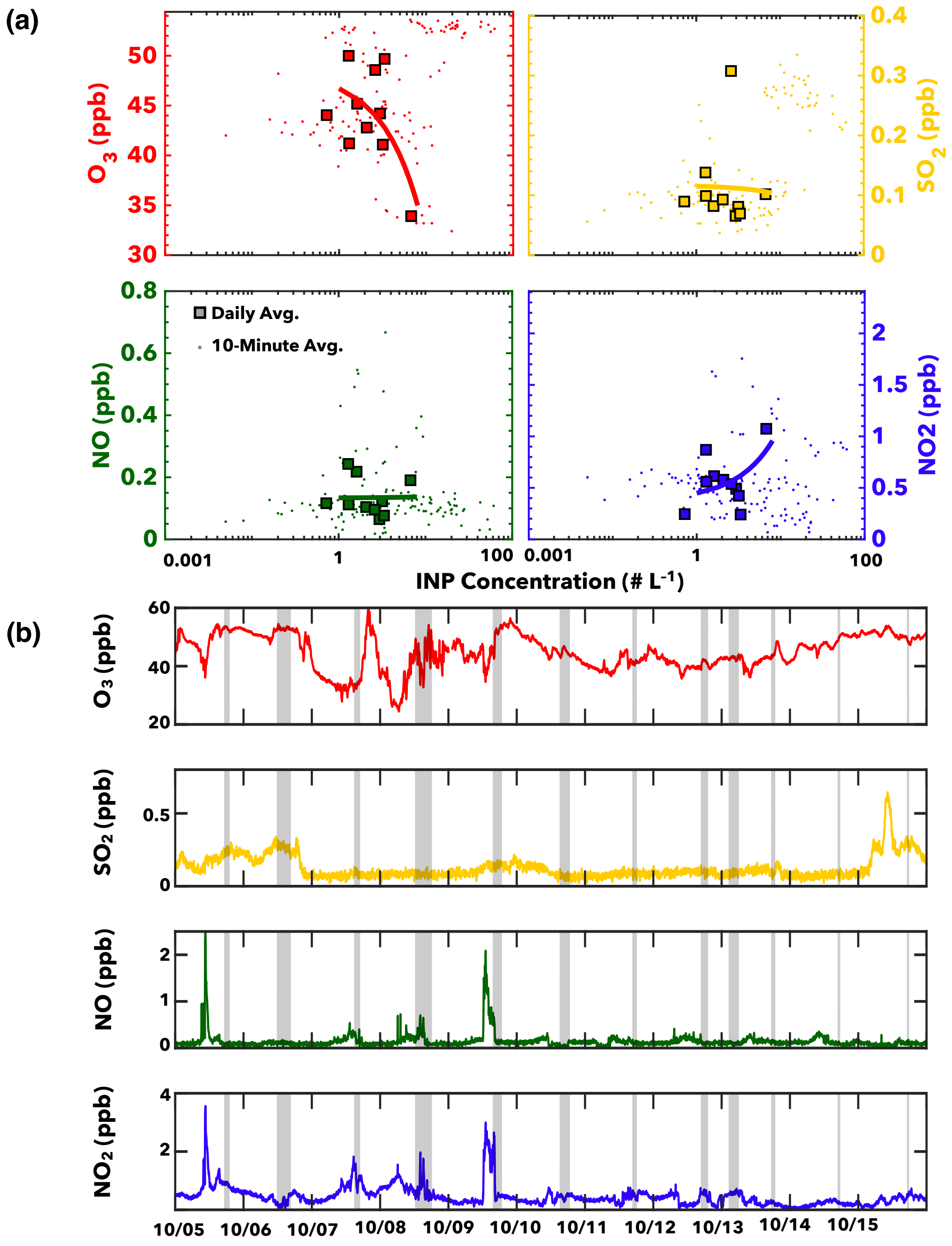

Supplementary Figure 4. Puy de Dôme observatory gas phase chemistry. **(a)** Variables plotted against INP concentrations. Each data point represents the average within a 10-minute or daily INP sampling period. Trendlines indicate linear regressions of daily averaged values. **(b)** Time series of data. Shaded regions correspond to periods of INP measurements.

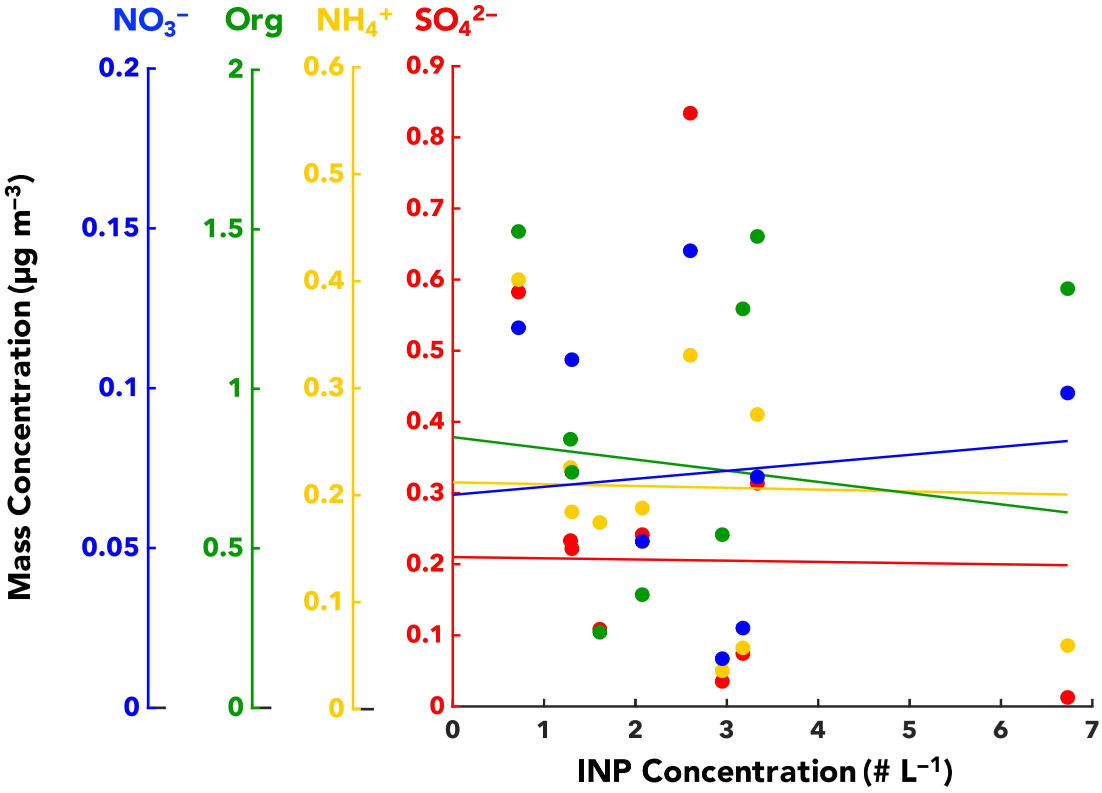

Supplementary Figure 5. Correlations between INP concentrations and bulk ambient $PM_{2.5}$ aerosol concentrations. Each data point represents daily averaged values of INP and ACSM aerosol mass concentrations. Also illustrated are ordinary least squares linear regressions of each relationship.

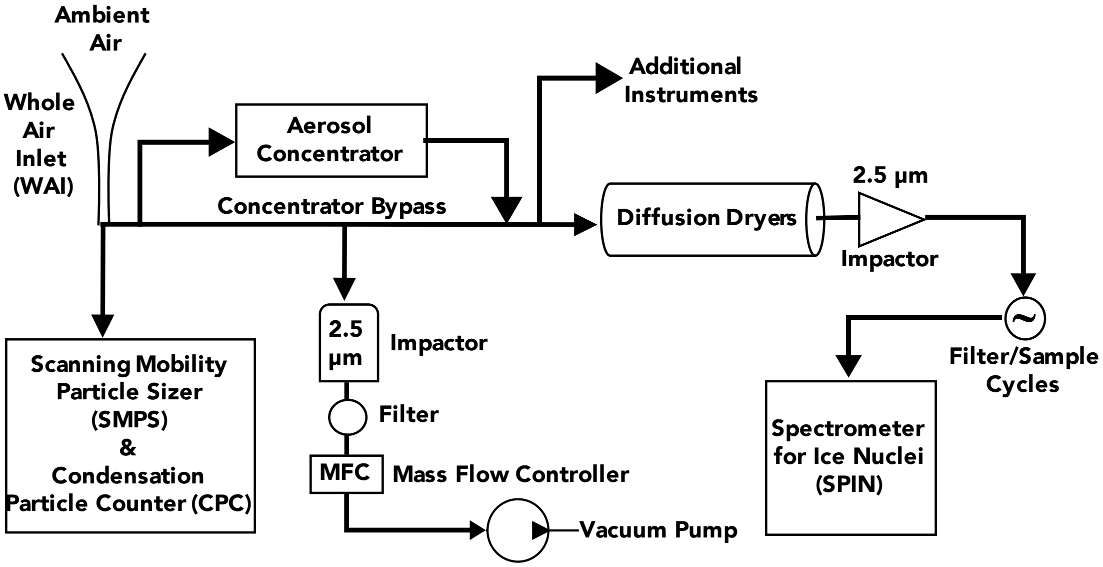

Supplementary Figure 6. Experimental Setup. Aerosols were sampled through a whole air inlet to evaporate cloud particles in the event of in-cloud sampling. The scanning mobility particle sizer (SMPS) and filter/substrate samples drew directly from the inlet. The spectrometer for ice nuclei (SPIN) alternated sampling from the aerosol particle concentrator and directly from the inlet. Other instruments participating in the Puy de Dôme Ice Nucleation Intercomparison Campaign (PICNIC) simultaneously sampled from the inlet or concentrator. Results from these instruments will be published elsewhere.

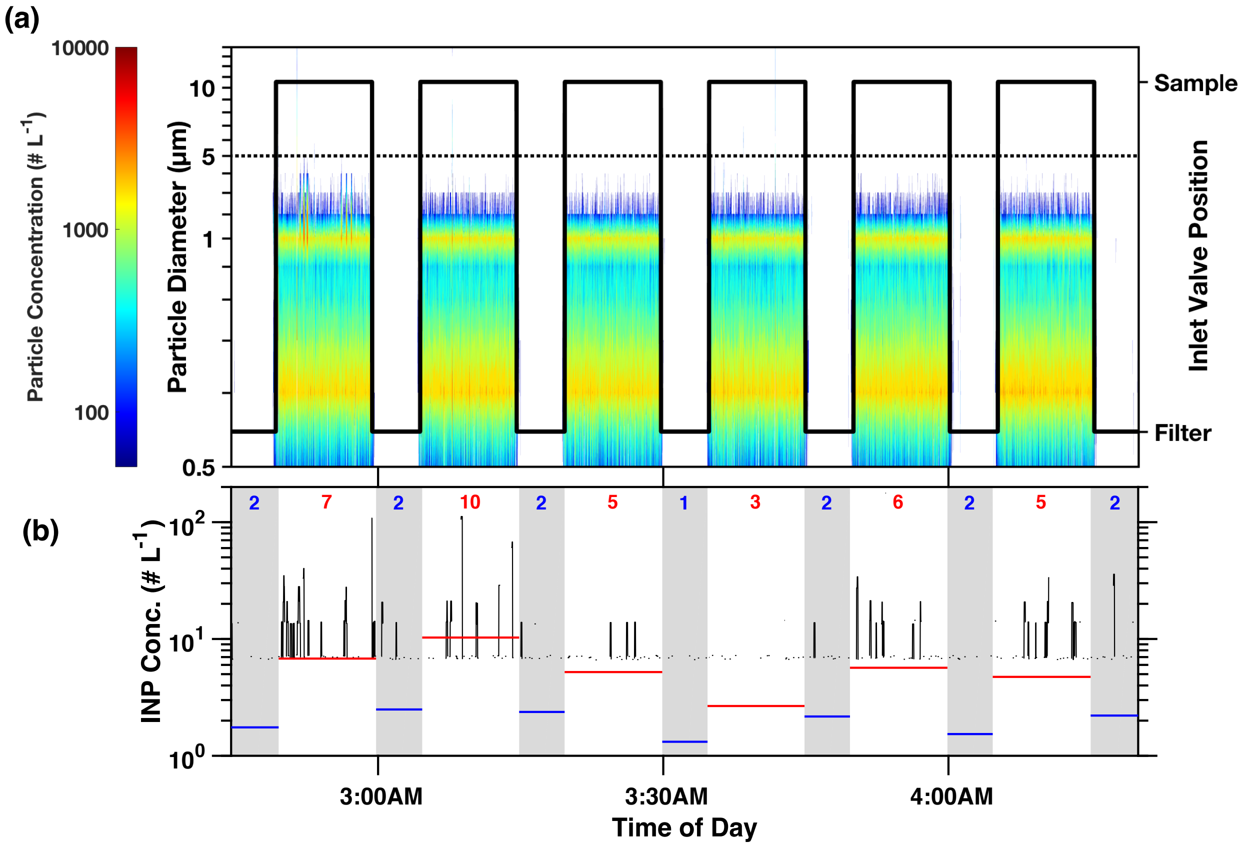

Supplementary Figure 7. Measuring ambient INP Concentrations. These data illustrate a sampling period off the aerosol concentrator on October 13th. (**a**) SPIN OPC time series measurements. The bimodality of the size distribution is due to the 1 µm size threshold of the high and low gain detectors on the OPC. Particles larger than 5 µm in diameter are counted as activated INPs. (**b**) INP concentration time series using the 5 µm size threshold. Solid blue lines show the average background concentrations during filter periods. Solid red lines show the average INP concentrations during measurement periods. These values are specified at the top of the panels.

Supplementary Tables

Supplementary Table 1. Regressions for INP Concentration and Meteorological Variables.

	**10-Minute Sampling**		**Daily Sampling**	
**Variable**	**R^2**	**p-value**	**R^2**	**p-value**
Temperature	0.027	0.134	0.431	0.039
Pressure	0.087	0.256	0.210	0.183
Relative Humidity	0.272	0.313	0.286	0.111
Wind Speed	0.096	0.207	0.027	0.648
Wind Direction	0.014	0.607	0.519	0.099
Cloud Liquid Water Content	0.021	0.142	0.279	0.117

Supplementary Table 2. Regressions for INP Correlations at Puy de Dôme.

**Variable**	**R^2**	**p-value**
[INP] vs. [$P_{D < 150\ nm}$]	0.880	1.93×10^{-5}
[INP] vs. [$P_{D > 150\ nm}$]	0.064	0.455
INP_D vs. Organic Mass %	0.684	0.002

Supplementary Table 3. Regression Coefficients for INP Concentration and Gas Phase Chemistry.

	**10-Minute Sampling**		**Daily Sampling**	
**Variable**	**R^2**	**p-value**	**R^2**	**p-value**
Ozone (O_3)	0.148	0.343	0.351	0.0713
Sulfur Dioxide (SO_2)	0.337	0.102	0.002	0.915
Nitric Oxide (NO)	0.028	0.520	0.001	0.973
Nitrogen Dioxide (NO_2)	0.001	0.684	0.236	0.154

Supplementary Table 4. Regression Coefficients for INP Concentration and Bulk Aerosol Composition.

**Variable**	**R^2**
Sulfate (SO_4^{2-})	1.1×10^{-4}
Ammonium (NH_4^+)	2.2×10^{-4}
Total Organics (Org)	0.011
Nitrate (NO_3^-)	0.0060

Supplementary References

1. Gute, E. *et al.* Field evaluation of a Portable Fine Particle Concentrator (PFPC) for ice nucleating particle measurements. *Aerosol Sci. Technol.* **53**, 1067–1078 (2019).
2. Zhang, Y. *et al.* The Cooling Rate- and Volatility-Dependent Glass-Forming Properties of Organic Aerosols Measured by Broadband Dielectric Spectroscopy. *Environ. Sci. Technol.* **53**, 12366–12378 (2019).
3. DeRieux, W.-S. W. *et al.* Predicting the glass transition temperature and viscosity of secondary organic material using molecular composition. *Atmos. Chem. Phys.* **18**, 6331–6351 (2018).
4. Riva, M. *et al.* Increasing Isoprene Epoxydiol-to-Inorganic Sulfate Aerosol Ratio Results in Extensive Conversion of Inorganic Sulfate to Organosulfur Forms: Implications for Aerosol Physicochemical Properties. *Environ. Sci. Technol.* **53**, 8682–8694 (2019).
5. Hansen, A. M. K. *et al.* Hygroscopic properties and cloud condensation nuclei activation of limonene-derived organosulfates and their mixtures with ammonium sulfate. *Atmos. Chem. Phys.* **15**, 14071–14089 (2015).
6. Angell, C. A. Formation of Glasses from Liquids and Biopolymers. *Science (80-.).* **267**, 1924–1935 (1995).
7. Renbaum-Wolff, L. *et al.* Viscosity of α-pinene secondary organic material and implications for particle growth and reactivity. *Proc. Natl. Acad. Sci. U. S. A.* **110**, 8014–9 (2013).
8. Knopf, D. A., Alpert, P. A. & Wang, B. The Role of Organic Aerosol in Atmospheric Ice Nucleation: A Review. *ACS Earth Sp. Chem.* **2**, 168–202 (2018).
